# Immune monitoring technology primer: whole exome sequencing for neoantigen discovery and precision oncology

**DOI:** 10.1186/s40425-016-0126-0

**Published:** 2016-04-19

**Authors:** Pia Kvistborg, Raphael Clynes, Wenru Song, Jianda Yuan

**Affiliations:** 1 Netherlands Cancer Institute, Postbus 90203, 1006 BE Amsterdam, Netherlands; 2 Bristol-Myers Squibb, 3551 Lawrenceville Road, Princeton, NJ 08648 USA; 3 AstraZeneca, One MedImmune Way, Gaithersburg, MD 20878 USA; 4 Oncology Clinical Research, Merck Research Laboratories, Rahway, 07065 NJ USA

**Keywords:** Immune monitoring, Whole exome sequencing, Biomarker, Neoantigen, Mutation load, Personalized cancer immunotherapy, Precision oncology

## Description of the technology

**Fig. 1:**
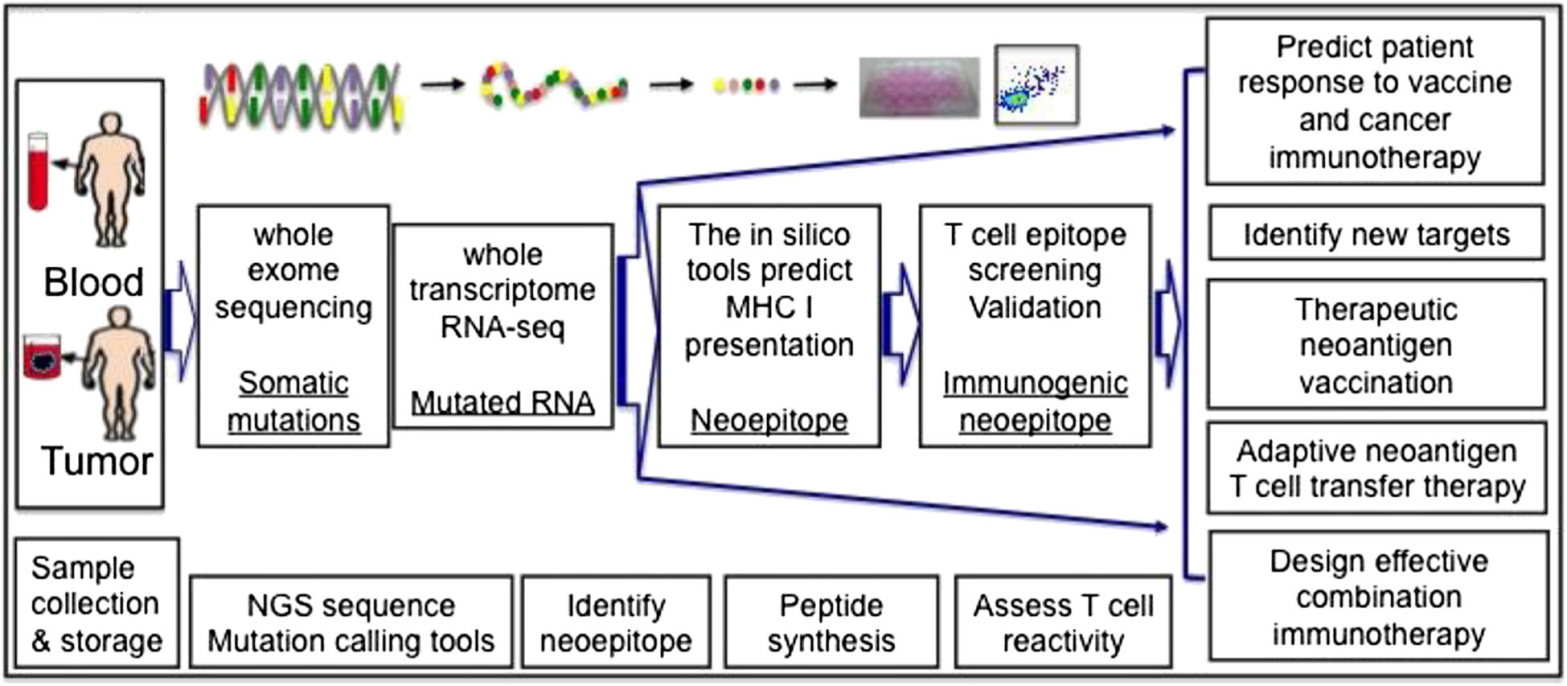
Current potential pipelines of whole exome sequencing for neoantigen discovery and precision oncology. After sample collection, whole exome sequencing can be performed on both tumor and non-transformed cells from the same patient. Once tumor specific mutations are identified, RNA-seq can be utilized to determine the level of expression of the mutations. Computational tools and/or a tandem minigene library are used to identify the neoepitopes, T cell assays to narrow down the true immunogenic neoepitope for efficient assessment and precise prediction and neoantigen vaccination targets. Neoantigen discovery also provides guidance for adaptive neoantigen T cell transfer therapy and combination immunotherapy

Tumor rejection antigens allow tumors sufficiently distinct from normal tissue to activate the immune system and induce an efficient anti-tumor response. Tumor mutated specific antigens (TMSA, neoantigens) without central tolerance are major tumor rejection antigens. The recent developments of innovative deep sequencing technologies (at an affordable cost) along with advances in bioinformatics have enabled systemic analysis of the mutation load of the tumor as well as identification of the potentially immunogenic neoantigens. T cell reactivity against these predicted neoantigens can then be analyzed [1, 2]. This novel approach allows the discovery of the mutated genes in individual tumors and assessment of the immunogenicity of these neoepitopes. It consists of several key steps as illustrated in Fig. 1, including a) sample collection and storage, b) whole exome sequencing to identify the mutations by using different computational and mutation calling tools, c) RNA-seq analysis to focus specifically on the expressed mutations, d) identification of neoepitopes in silico with computational algorithms for MHC class I and class II binding as well as e) use of tandem minigene libraries for class II epitope screening and f) neoantigen specific T cell assays to differentiate trueimmunogenic neoepitopes from putative ones. Tumor and non-transformed cells (usually PBMCs) from the same patients can be sequenced to determine the mutation load and the full range of genomic alterations within a tumor, such as nucleotide substitutions, structural rearrangements and copy number alterations. The data to date indicate that the vast majority of mutated antigens are not shared between patients, and are considered patient-specific [1]. The genetic landscape and the full spectrum of genomic alterations in each individual tumor provide potential guidance for personalized cancer immunotherapy and precision oncology.

## Type of data obtained/readout

Deep sequencing to assess the mutations present within the protein-encoding regions of the genome (the exome) of an individual tumor will generate a unique set of data for each tumor. Whole exome sequencing data from the tumor sample and non-transformed cells will be used to detect nonsynonymous somatic mutations with the use of mutation calling tools. RNA seq analysis will be used to identify expressed mutations in order to predict potential neoantigens. Epitope prediction algorithms based on published or submitted MHC Class I and II binding data will provide estimates of binding affinity to identify putative T cell neoepitopes. Data resulting from functional assays, including combinatorial encoding of MHC multimer screening flow cytometry assays, or functional read outs such as cytokine production, will provide an indication of T cell reactivity to validate the tumor-specific immunogenic neoepitopes. The analyses of mutations in MHC class I and II genes as well as key molecules affecting antigen processing and presentation are vital to provide a better assessment of their potential impact on cytolytic T cell responses. The genetic landscape, the pool of neoepitopes and functional tumor rejection measures of neoantigen-specific T cells (tumor recognition) could be used to further assess their relevance to clinical outcome, design therapeutic tumor-specific neoantigen (TSNA) vaccination, apply adoptive neoantigen T cell transfer therapy and to guide more effective immuno-oncology combination immunotherapy.

## Limitations of the approach

One of the major limitations of this approach is in the early stage computational tools that are used both to identify tumor-specific mutations and to guide epitope prediction. Multiple computational tools, such as EBcall, JointSNVMix, MuTect, SomtaticSniper, Strelka and VarScan 2, are used to compare tumor samples to normal tissue at each variant locus to increase the accuracy of somatic single nucleotide variant (sSNV) calling [3–7]. Because these tools use distinct variant calling algorithms, there may be variability in the somatic mutations identified. Thus, more validation studies are necessary to improve the calling tools and standardize their use. Computer algorithm-guided epitope prediction and the tandem minigene library approach are used to identify MHC Class I or II binding neoepitopes recognized by neoantigen specific CD8^+^ and CD4^+^ T cells, respectively [8–10]. The accuracy of the prediction algorithms mostly depends upon the binding scores to the MHC complex, with the Class II prediction tools being much less well-developed than Class I. Tumors, especially those with mutant and viral antigens, could be sufficiently “foreign” to be recognized by the immune system. However, current data has illustrated that autologous T cells did not recognize the vast majority of neoepitopes. Although the epitope prediction tools have been shown to have a high degree of overlap [11–14], it is important to improve the ability of these tools to differentiate putative neoepitopes from real immunogenic neoepitopes [15]. This lack of immunogenicity could also be due to the tumor’s inability to activate the immune system because of additional resistance mechanisms, especially tumor microenvironment factors, rather than the absence of tumor antigens. Because the activation and cytotoxic signals in individual tumors may reflect the overall status of a neoantigen-specific tumor response, it will be critical to further evaluate these functional signatures and to incorporate them into future optimized pipelines.

Another potential limitation of this technology is that representative, high-quality tissue samples are needed in order to produce reliable results. Tumor tissue from formalin-fixed, paraffin-embedded (FFPE) samples may be used for whole exome sequencing. However, proper collection and storage of the tumor tissue is essential to ensure high quality DNA for deep sequencing. Because of the heterogeneity of the tumor, it is also essential to collect representative tissue to avoid any bias. In addition, mutational profiles may change due to disease progression or ongoing treatment. Therefore, assessing the tumor sample closest to the intervention is best to eliminate the potential variation and increase accuracy. Moreover, although PBMCs are commonly used as non-transformed cells, the signal from even low frequency circulating tumor cells from whole blood needs to be further validated for potential contribution to data noise.

## Types of samples needed and special issues pertaining to samples

Tissue from the tumor sample and non-transformed cells are needed for whole exome sequencing. However, as mentioned above, proper collection and storage of representative tissue is essential to ensure high-quality samples for deep sequencing. For downstream assessment of T cell reactivity in functional assays, TILs and PBMCs are needed and must be viably preserved as a single-cell suspension.

## Level of evidence

This is a novel technology that is still currently under development. Two pilot preclinical studies in mouse models first demonstrated that whole exome sequencing is efficient to identify neoantigen-specific CD8+ T cells with tumor elimination [16, 17]. Several human clinical studies highlighted the feasibility and importance of understanding the immunogenicity of neoantigens and their potential clinical application in patients treated with tumor-infiltrating lymphocyte cells [8–10]. The level of mutational load (or the mutational landscape) as a potential biomarker was associated with clinical outcome to immune checkpoint blockade cancer immunotherapy in patients with advanced melanoma, non-small cell lung cancer (NSCLC) and colorectal cancer [18–21]. Patients with highly mutagenized tumors and activated cytolytic markers are most likely to respond to checkpoint blockade treatment [22]. In this study, epitope prediction did not improve clinical outcome prediction value [23]. However, some patients with a high mutational load do not experience clinical responses, while some patients with a low mutation profile experience substantial clinical responses [18, 19]. Assessment of clinically relevant immunogenic mutation loads along with active cytolytic signatures before therapy is pivotal to improve the accuracy of outcome prediction. As the study was performed in patients with mismatched repair deficiency tumors [20], more prospective studies must be performed to determine whether the mutation load can guide novel therapeutic approaches to selectively enhance T cell response to neoantigens in future mono- or combination therapies.

## Abbreviations

DNA: deoxyribonucleic acid
FFPE: formalin-fixed, paraffin-embedded (tissue)
MHC: major histocompatibility complex
NGS: next-generation sequencing
NSCLC: non-small cell lung cancer
PBMCs: peripheral blood mononuclear cells
RNA: ribonucleic acid
sSNV: somatic single nucleotide variant
TILs: tumor infiltrating lymphocytes
TMSA: tumor mutation specific antigen
TSNA: tumor specific neoantigens
